# Supplementary material for: Electrically-controlled digital metasurface device for light projection displays
Source: Nat Commun. 2020 Jul 17;11:3574. doi: 10.1038/s41467-020-17390-3 (PMC7367846; doi:10.1038/s41467-020-17390-3)
Supplement: Supplementary file 1 — Supplementary Information [file 41467_2020_17390_MOESM1_ESM.pdf]

Supplementary Materials for

Electrically-controlled digital metasurface device for light projection displays

Jianxiong Li[1], Ping Yu[1], Shuang Zhang[2], and Na Liu[3,4]*

[1]Max Planck Institute for Intelligent Systems, Heisenbergstrasse 3, 70569 Stuttgart, Germany
[2]School of Physics & Astronomy, University of Birmingham, Birmingham B15 2TT, UK.
[3]Kirchhoff Institute for Physics and Centre for Advanced Materials, University of Heidelberg, Im Neuenheimer Feld 227, 69120 Heidelberg, Germany.
[4]Max Planck Institute for Solid State Research, Heisenbergstrasse 1, 70569 Stuttgart, Germany
*e-mail: na.liu@kip.uni-heidelberg.de

Design of the metasurface holograms

To generate a target image, a phase-only hologram with a unit cell size of 600 nm was designed based on the Gerchberg-Saxton algorithm. The off-axis angle information (θ_x, θ_y) of the seven holographic segments in Fig. 4 from main text is shown in Supplementary Fig. 7b. The off-axis angle of the reconstructed hologram in Fig. 5 from main text was (0°, 11°). Owing to the large angular range, the Rayleigh-Sommerfeld diffraction method was used to simulate the holographic image. The hologram was precompensated to avoid pattern distortions.

Light output of the optical device

We consider a travelling plane wave incident from the *z*-direction, which is then reflected by an optical device. The reflection process can be described as follows:

$$g(x,y,z) = H(v_x,v_y)\,f(z) = A\exp[-j2\pi(v_x x + v_y y)]\exp(-jk_z z),$$
$$k_z = \sqrt{k^2 - k_x^2 - k_y^2} = 2\pi\sqrt{\lambda^{-2} - v_x^2 - v_y^2},$$

where *f(z)* and *g(x, y, z)* are the wave functions of the input and output light, respectively. $H(v_x, v_y)$ is the transfer function of the optical device. *A* is a constant. *k* and λ are the light wavevector and wavelength, respectively. $v_x = k_x/2\pi$ and $v_y = k_y/2\pi$ correspond to the spatial frequencies of the optical device along the *x*- and *y*-directions, respectively. The circle defined by $v_x^2 + v_y^2 = \lambda^{-2}$ thus separates the zones of output propagating (grey) and evanescent (white) waves in the two-dimensional spatial frequency spectrum as illustrated in Fig. 2a from main text.

Polarization dependence

When the incident light is linearly polarized along the long-axis of the LC molecules at V = 0 (see Supplementary Fig. 5b), the polarization of light does not change after transmitting through the LC layer. Based on the Pancharatnam-Berry phase, two centrosymmetric holographic images occur on the left and right sides with respect to the location of the zero-order reflected light simultaneously[1] (see Supplementary Fig. 5e). At V = 20V, both of the images are switched off. When the polarization of the incident light is 45° relative to the long-axis of the LC molecules, and the thickness d of the LC layer satisfies $d = \lambda(1/4+m)/(n_o-n_e)$, the incident light is converted to circular polarization. n_o and n_e represent the ordinary and extraordinary refractive indices of the LCs, respectively. λ is the incident light wavelength and m is an integer. Thus, only one switchable holographic image occurs (see Supplementary Fig. 5d).

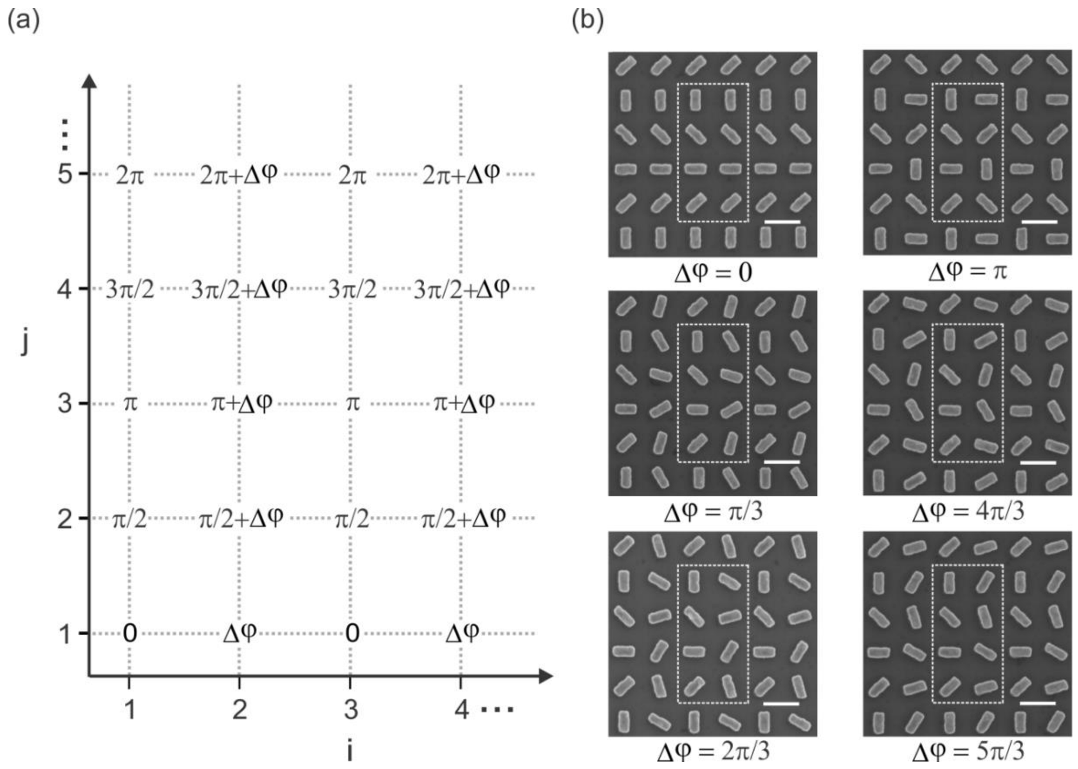

Supplementary Fig. 1 Phase profiles of the metasurfaces. (a) Discrete phase distribution profile defined in Eq. 1. (b) SEM images of the corresponding metasurfaces with different values of $\Delta\varphi$ for Fig. 2c.

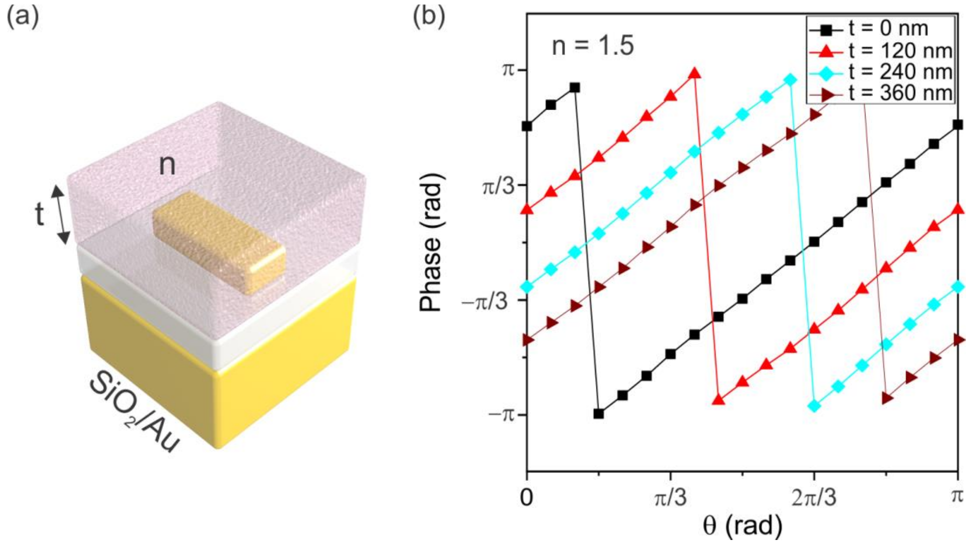

Supplementary Fig. 2 Modulation of the geometric and propagation phases. (a) Schematic of the metasurface unit cell (300 nm × 300 nm) covered by a dielectric material with refractive index of n = 1.5 and thickness of t. The gold nanorod has a dimension of 200 nm × 80 nm × 30 nm. It resides on a gold mirror spaced by SiO_2 (100 nm). (b) Simulated phase profile in dependence on the gold nanorod orientation θ and dielectric spacer thickness t.

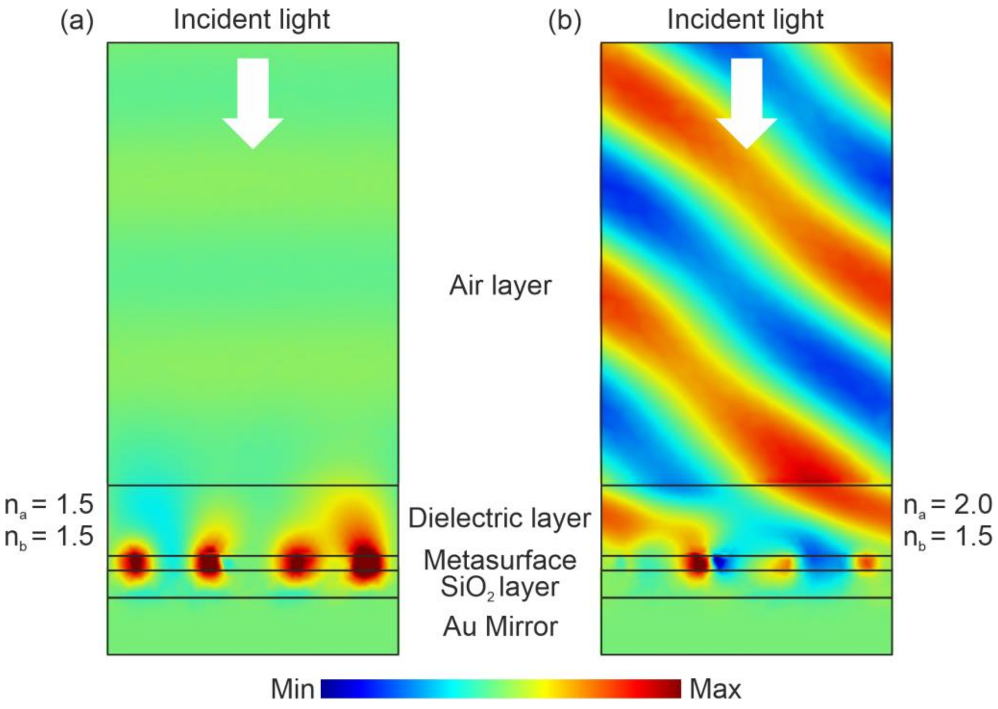

Supplementary Fig. 3 Simulated electric field distributions of the anomalously reflected light. (a) $n_a = n_b = 1.5$. (b) $n_a = 2.0$, $n_b = 1.5$.

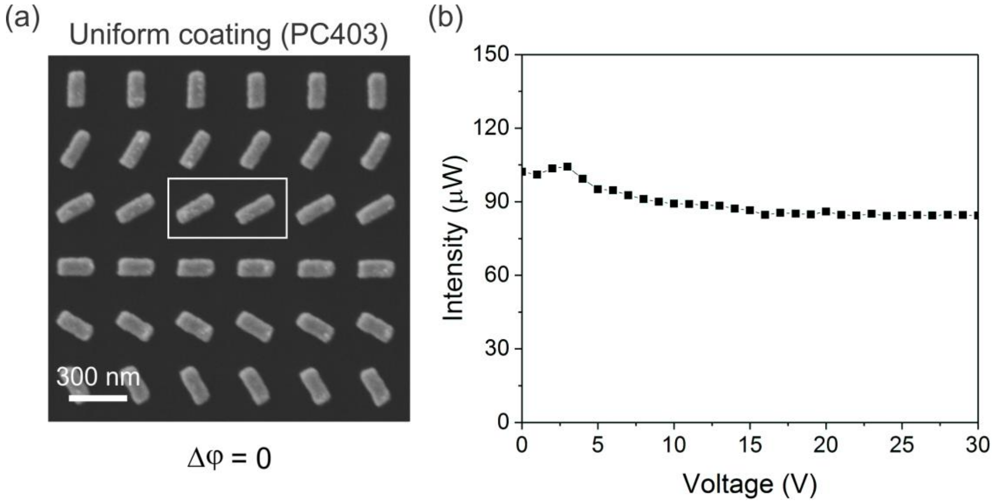

Supplementary Fig. 4 Performance of the metasurface without PMMA trenches. (a) SEM image of the metasurface with uniform coating (PC403). (b) Intensity of the anomalously reflected light as a function of the applied voltage V. There is no significant change of the intensity, when increasing the applied voltage.

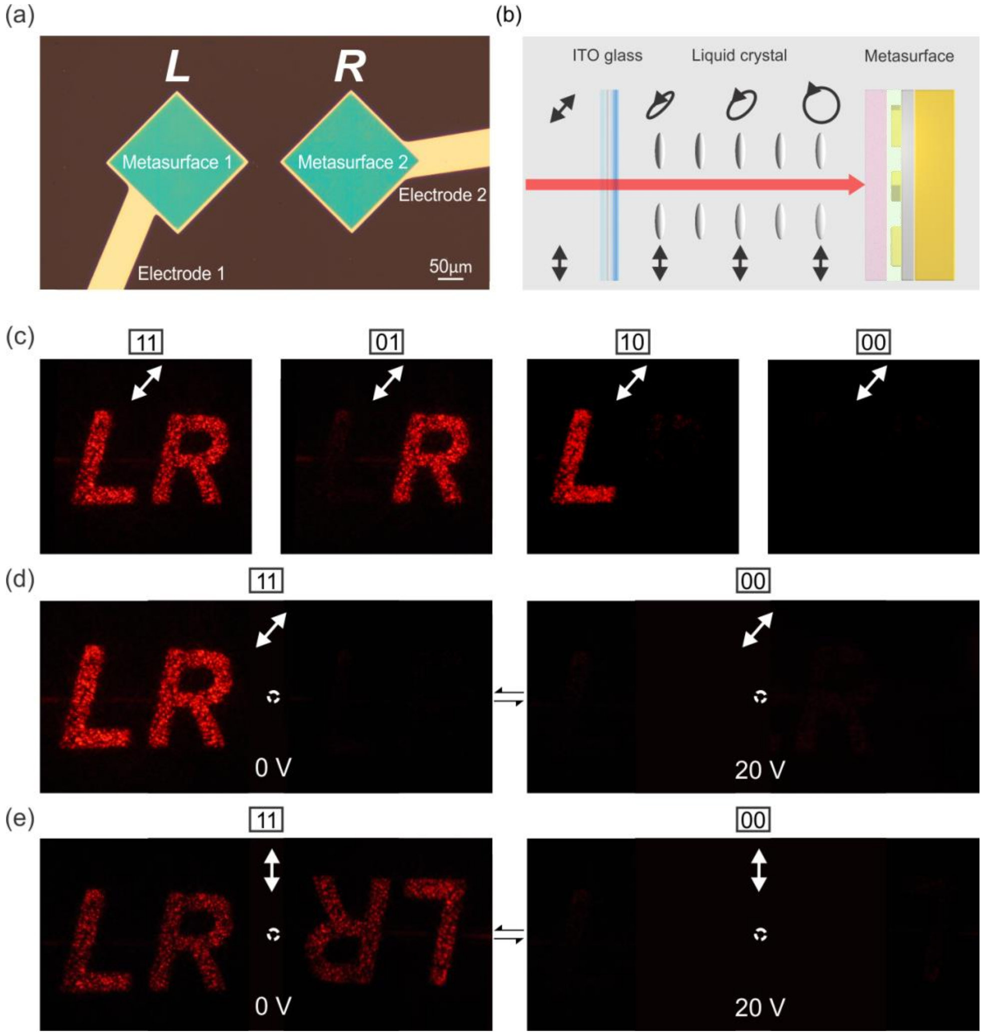

Supplementary Fig. 5 Polarization dependence of the hologram. (a) Photograph of the electrically-controlled DMSD for arbitrary dynamic holographic pattern generation. There are two metasurface pixels 1 and 2 on the device, which are independently controlled by electrodes 1 and 2, respectively. (b) Schematic of the interactions between the incident light with different linear polarization directions and the LC layer. (c) Two holographic patterns 'L' and 'R' are independently switched on and off without crosstalk. (d) Experimental result of the dynamic holographic images generated on the left and right sides, when the direction of the linearly polarized light is 45° relative to the long-axis of the LC molecules. (e) Experimental result for the case, when the direction of the linearly polarized light is incident along the long-axis of the LC molecules.

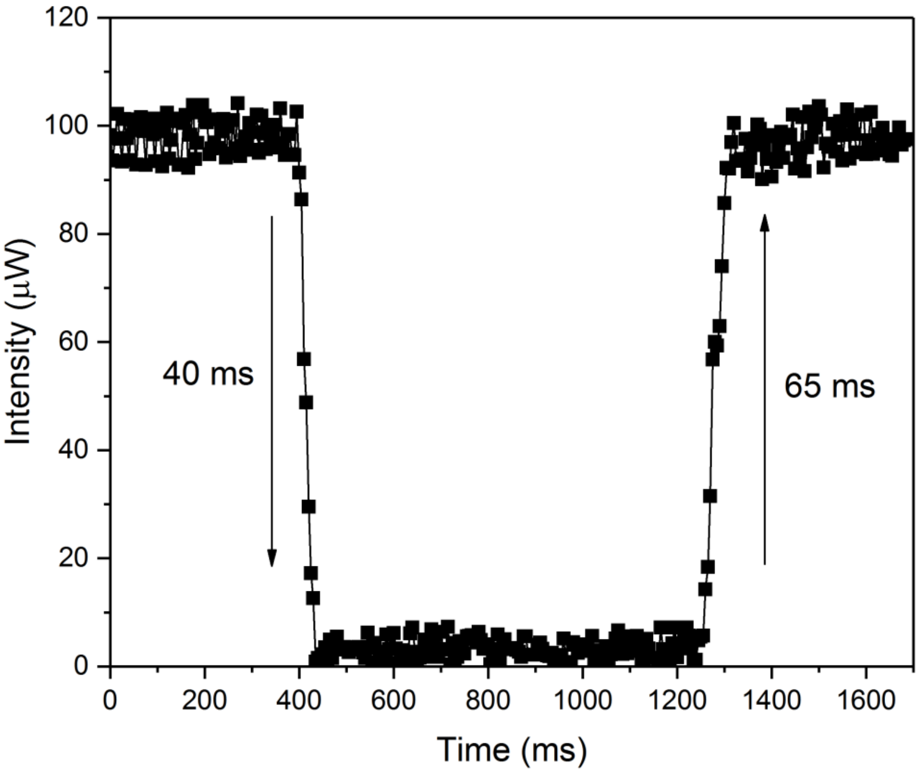

Supplementary Fig. 6 Switching rate characterization. The switching time of 65 ms and 40 ms is achieved for on and off states, respectively.

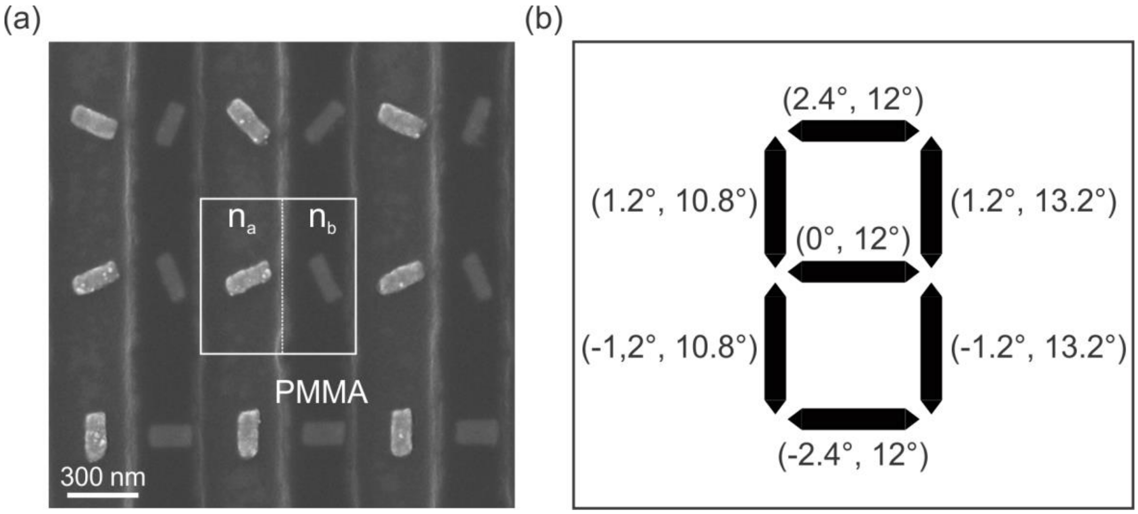

Supplementary Fig. 7 Design of the metasurface for the numeric indicator display. (a) SEM image of the metasurface pixel (M4) in Fig. 4b from main text . The gold nanorods in the neighboring columns are orthogonally oriented as highlighted in the white frame. The periodicities along both directions are 600 nm. In each pair, one nanorod is covered by PMMA and the other nanorod is employed to yield the required phase profile for generating the desired

holographic pattern. (b) Off-axis angle information (θ_x, θ_y) of the seven holographic segments in Fig. 4 from main text.

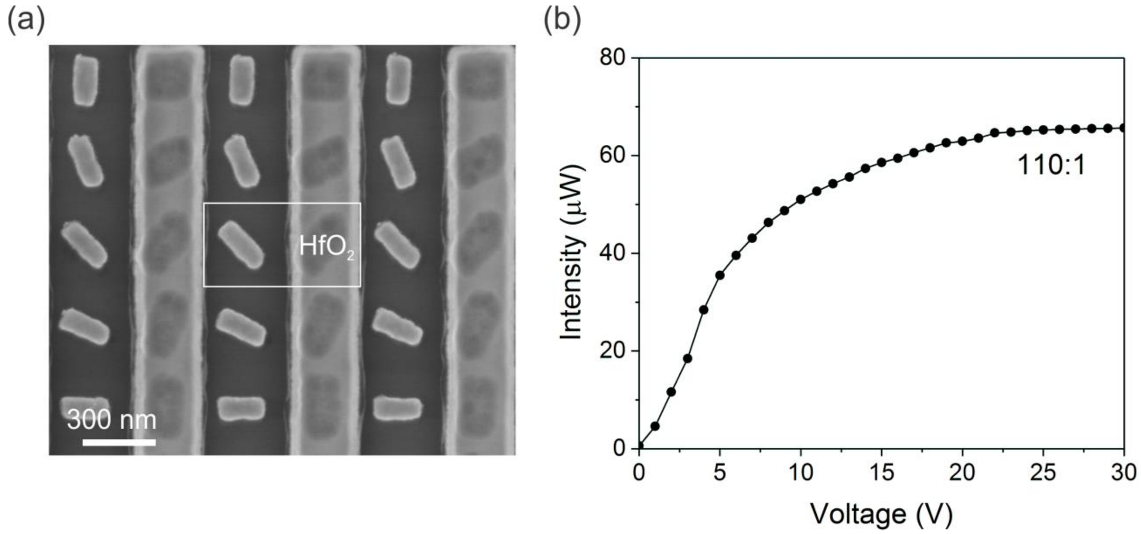

Supplementary Fig. 8 Switching performance of the metasurface with selective HfO_2 coating. (a) SEM image of the metasurface with selective HfO_2 coating. (b) Intensity of the anomalously reflected light as a function of the applied voltage V. An intensity modulation ratio as large as 110:1 is achieved.

References

1 Wen, D. et al. Helicity multiplexed broadband metasurface holograms. *Nat. Commun.* **6**, 8241 (2015).
