# Supplementary material for: Electrically-controlled digital metasurface device for light projection displays
Source: Nat Commun. 2020 Jul 17;11:3574. doi: 10.1038/s41467-020-17390-3 (PMC7367846; doi:10.1038/s41467-020-17390-3)
Supplement: Supplementary file 2 — Description of Additional Supplementary Files [file 41467_2020_17390_MOESM2_ESM.pdf]

Description of Additional Supplementary Files

File Name: Supplementary Movie 1
Description: Dynamic switching of the 4-bit DMSD

File Name: Supplementary Movie 2
Description: Dynamic switching of numeric indicator display

File Name: Supplementary Movie 3
Description: Dynamic switching of electrically-controlled dynamic holograms
